# Complete Genome Sequence of *Sphingomonas* sp. Strain NIBR02145

**DOI:** 10.1128/mra.00067-23

**Published:** 2023-06-22

**Authors:** Hyun Young Kim, Jin Young Park, Jin Nam Kim, Jaewoong Yu, Bongsang Kim

**Affiliations:** a Department of Oral Microbiology and Immunology, School of Dentistry, Seoul National University, Seoul, Republic of Korea; b Dental Research Institute, School of Dentistry, Seoul National University, Seoul, Republic of Korea; c Biological Resources Research Department, National Institute of Biological Resources, Incheon, Republic of Korea; d eGnome, Inc, Seoul, Republic of Korea; e Department of Agricultural Biotechnology, Seoul National University, Seoul, Republic of Korea; f Research Institute of Agriculture and Life Sciences, Seoul National University, Seoul, Republic of Korea; University of Southern California

## Abstract

*Sphingomonas* sp. strain NIBR02145 is a putative chemoheterotrophic strain that was isolated from soil in Wando-gun, Republic of Korea. The NIBR02145 genome was sequenced with PacBio next-generation sequencing technology. The 5,010,245-bp circular genome has a GC content of 66.79% and harbors 4,561 coding sequences, 6 rRNAs, and 52 tRNAs.

## ANNOUNCEMENT

The genus *Sphingomonas* was first proposed in 1990 by Yabuuchi et al. (1). The strains of the genus are Gram-negative, strictly aerobic, chemoheterotrophic bacteria (2, 3). *Sphingomonas* strains are found in a variety of habitats, including soil, water, plant roots, and even extreme environments (4–6). They are attracting great attention due to their properties that can be applied to biotechnology, such as biosynthetic abilities (7, 8) and biodegrading abilities (9). Therefore, whole-genome-level study of *Sphingomonas* strains is expected to make significant contributions not only to the possibility of industrial applications but also to ecological research. In addition, although there are 3,960 *Sphingomonas* taxonomies in NCBI, only 139 of them have genome data, which limits comparative and phylogenetic analyses of *Sphingomonas* strains.

A sample of topsoil was obtained at a depth of <10 cm in Wando-gun, Jeollanam-do, Republic of Korea (34°18′52.4″N, 126°42′50.5″E). Strain NIBR02145 was cultured in Reasoner’s 2A (R2A) medium (Merck, Millipore, Germany) and incubated at 25°C for 3 days, and cells from a single colony were collected in a 5-mL Eppendorf tube for DNA extraction. Genomic DNA was extracted using a MagCore genomic DNA bacterial kit (RBC Bioscience, Taiwan), and a Covaris g-TUBE device was used to shear the DNA according to the instructions of the manufacturer. DNA was sheared into fragments of >15 kb and purified using 0.45× AMPure XP magnetic beads (Beckman Coulter, USA). Library construction was performed using the Pacific Biosciences (PacBio) SMRTbell library preparation kit v1.0 (PacBio). DNA damage repair and end repair were performed using the SMRTbell damage repair kit (PacBio), and then reagents from the SMRTbell enzyme cleanup kit (PacBio) were added to the library. The library was placed in 0.75% BluePippin and electrophoresed to obtain fragments of ≥15 kb, and then the library was sequenced on the PacBio RS II platform.

The number of sequenced raw reads was 1,747,074 (14,482,769,372 bp), and the *N*_50_ value was 9,716 bp. Reads of <1,000 bp were filtered using SeqKit v2.3.0, and then *de novo* assembly was conducted with Flye v2.8 (10). The assembly resulted in one circular complete genome of 5,010,245 bp, with a GC content of 66.79%. The quality of the genome assembly was assessed with Benchmarking Universal Single-Copy Orthologs (BUSCO) v5.2.2 with the bacteria_odb data set (11). The result showed that 123 of the 124 BUSCOs were complete single-copy BUSCOs. The genome was annotated using the NCBI Prokaryotic Genome Annotation Pipeline (PGAP) v6.5 (12). The genome of NIBR02145 contained 4,561 coding sequences, 6 rRNAs, 52 tRNAs, and 3 noncoding RNAs (ncRNAs). The statistical information for the genome sequence of NIBR02145 is presented in Table 1. The 16S rRNA gene of NIBR02145 was compared with the NCBI 16S rRNA gene database using BLASTn (13). In the BLASTn comparison, the 16S rRNA gene of Sphingomonas kyeonggiensis THG-DT81 (GenBank accession number NR_134182.1) showed the greatest similarity (98.5%). In addition, average nucleotide identity (ANI) values were calculated with pyani v0.2.12 (14). The greatest ANI value was 87.3% for S. kyeonggiensis THG-DT81. Default parameters were used for all software unless otherwise specified. All of these results suggested that NIBR02145 is closely related to Sphingomonas kyeonggiensis.

**TABLE 1:** Statistical information for the genome sequence of *Sphingomonas* sp. strain NIBR02145

Parameter	Value
No. of PacBio reads (>1,000 bp)	1,712,399
Genome coverage (×)	2,755
Genome size (bp)	5,010,245
No. of contigs	1
GC content (%)	66.79
No. of coding sequences	4,561
No. of tRNAs	52
No. of rRNAs	6
No. of ncRNAs	3

### Data availability.

The complete genome sequence of *Sphingomonas* sp. strain NIBR02145 has been deposited in GenBank under the accession number CP114791. The raw data have been deposited in the SRA under the accession number SRR22805145.
